# Discovery of Prenyltransferase-Guided Hydroxyphenylacetic Acid Derivatives from Marine Fungus *Penicillium* sp. W21C371

**DOI:** 10.3390/md22070296

**Published:** 2024-06-26

**Authors:** Cancan Wang, Ye Fan, Chenjie Wang, Jing Tang, Yixian Qiu, Keren Xu, Yingjia Ding, Ying Liu, Youmin Ying, Hong Wang

**Affiliations:** College of Pharmaceutical Science & Collaborative Innovation Center of Yangtze River Delta Region Green Pharmaceuticals, Zhejiang University of Technology, Hangzhou 310014, China; cancanw1120@163.com (C.W.); m18767175381_1@163.com (Y.F.); 211123070063@zjut.edu.cn (C.W.); t_joyce@126.com (J.T.); 13588779931@163.com (Y.Q.); 201805150118@zjut.edu.cn (K.X.); 19857445690@163.com (Y.D.); 15844357993@163.com (Y.L.)

**Keywords:** marine fungi, *Penicillium*, genome mining, phenylacetic acid, *β*-glucuronidase, bioactivity

## Abstract

Traditional isolation methods often lead to the rediscovery of known natural products. In contrast, genome mining strategies are considered effective for the continual discovery of new natural products. In this study, we discovered a unique prenyltransferase (PT) through genome mining, capable of catalyzing the transfer of a prenyl group to an aromatic nucleus to form C-C or C-O bonds. A pair of new hydroxyphenylacetic acid derivative enantiomers with prenyl units, (±)-peniprenydiol A (**1**), along with 16 known compounds (**2**–**17**), were isolated from a marine fungus, *Penicillium* sp. W21C371. The separation of **1** using chiral HPLC led to the isolation of the enantiomers **1a** and **1b**. Their structures were established on the basis of extensive spectroscopic analysis, including 1D, 2D NMR and HRESIMS. The absolute configurations of the new compounds were determined by a modified Mosher method. A plausible biosynthetic pathway for **1** was deduced, facilitated by PT catalysis. In the in vitro assay, **2** and **3** showed promising inhibitory activity against *Escherichia coli β*-glucuronidase (EcGUS), with IC_50_ values of 44.60 ± 0.84 μM and 21.60 ± 0.76 μM, respectively, compared to the positive control, *D*-saccharic acid 1,4-lactone hydrate (DSL). This study demonstrates the advantages of genome mining in the rational acquisition of new natural products.

## 1. Introduction

The secondary metabolites produced by filamentous fungi are crucial sources of valuable pharmaceuticals and agrochemicals. *Penicillium*, one of the most common fungal genera, consists of more than 350 species with a worldwide distribution [1]. These fungi colonize a wide range of habitats, including conventional environments such as soil, vegetation, food, and indoor spaces, as well as extreme environments on Earth [2]. The diverse lifestyles and habitats of *Penicillium* species equip them with robust metabolic capabilities. Since the discovery of penicillin from *P. rubens* (often identified as *P. chrysogenum*), the fungi of the genus *Penicillium* have been extensively studied for their production of bioactive secondary metabolites [3]. Over the past several decades, many secondary metabolites with diverse structures and intriguing biological activity have been discovered from *Penicillium*, making it a promising reservoir of novel drug leads. However, the metabolic potential of *Penicillium* is reported to be underestimated and remains to be fully exploited, despite being one of the most well-studied fungal genera. In a recent large-scale genome-based study, Nielsen and co-workers conducted the first genus-wide analysis of the genomic diversity of 24 *Penicillium* species and identified 1317 putative biosynthetic gene clusters (BGCs), highlighting the potential of these species as sources of new antibiotics and other pharmaceuticals [4].

As the largest biome on Earth, the marine ecosystem boasts extraordinarily rich biodiversity due to its extreme physical and chemical conditions compared to the terrestrial ecosystems [5]. This vast biodiversity is partly demonstrated by the unique groups of marine microorganisms that produce a variety of secondary metabolites [6]. A significant number of fungi inhabit the marine environment, found in sea water, sediments, or living organisms such as sponges, corals, and algae. Among these, marine fungi of the genus *Penicillium* have garnered considerable attention as an important source of secondary metabolites, featuring novel structures and remarkable bioactive properties [7]. To date, more than 500 novel natural products have been isolated and characterized from marine *Penicillium* fungi, exhibiting benefits such as antibacterial [8,9,10,11,12], anti-inflammatory [13], enzyme inhibition, and cytotoxic properties [14,15].

*Escherichia coli β*-glucuronidase (EcGUS) is one of the most abundant bacterial *β*-glucuronidases, catalyzing the hydrolysis of glucuronide conjugates to produce the corresponding aglycones [16]. Anticancer drug irinotecan and anti-inflammatory drug indomethacin produce glucuronidated metabolites that are rapidly hydrolyzed by EcGUS, resulting in highly toxic aglycones. The accumulation of these aglycones in the intestine can lead to severe gastrointestinal adverse reactions, such as fatal diarrhea [17]. Consequently, targeting EcGUS has emerged as a crucial strategy to mitigate the adverse gastrointestinal effects associated with these drugs.

**Figure 1:**
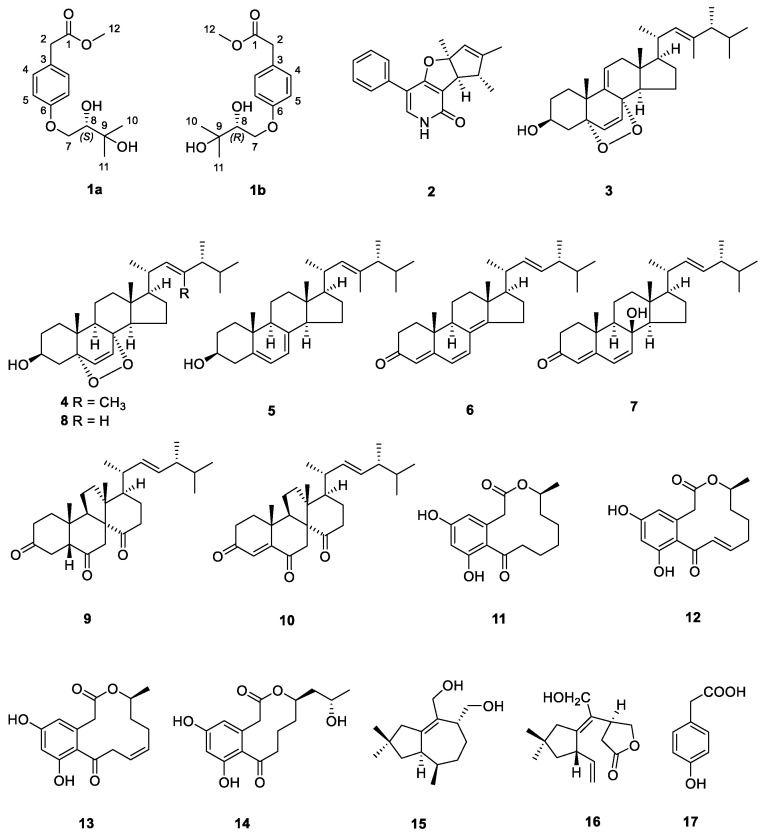
Chemical structures of compounds **1**–**17**.

The genome mining technique for the discovery of natural products, which leverages genomic sequence data to identify previously uncharacterized compounds, has proven effective and efficient for over a decade [18]. With the rapid accumulation of genomic sequences from various organisms, genome mining methods have led to the identification of an increasing number of natural products [19]. Traditional approaches often result in the rediscovery of known compounds, whereas genome-based strategies are now seen as a promising solution for the continual discovery of novel natural products. Prenyltransferases (PTs) are capable of catalyzing the transfer of prenyl groups from various prenyl donors. These prenylated secondary metabolites, including indole alkaloids, flavonoids, coumarins, ketones, quinones, and naphthalenes, are widely found in terrestrial and marine organisms and exhibit various types of biological activity, such as cytotoxicity, antioxidation, and antimicrobial effects [20]. For example, XptB, a ketone prenyltransferase from *Aspergillus nidulans*, catalyzes the *O*-prenylation of 1,7-dihydroxy-6-methyl-8-hydroxymethylxanthone, resulting in the production of variecoxanthone A [21]. Additionally, *Streptomyces* sp. CNQ-509 produces the rare *O*-prenylated phenazines, marinophenazines A and B, through the catalytic action of the enzyme CnqPT1 [22]. In our study, we analyzed the genomic data of PTs from our lab and the NCBI public databases, generated a sequence similarity network (SSN) of PTs, and discovered a previously uncharacterized branch. Phylogenetic tree analysis revealed that this enzyme belongs to a distinct branch of PTs, highlighting its unique characteristics. The secondary metabolites produced were investigated under different culture conditions using PT-guided isolation. A pair of new *p*-hydroxyphenylacetic acid derivative enantiomers, (±)-peniprenydiol A (**1**), containing prenyl units, and 16 known compounds (**2**–**17**) were isolated from *Penicillium* sp. W21C371 (Figure 1). Their structures were determined using thorough NMR analysis and a modified Mosher method. A plausible biosynthetic pathway for **1** was deduced, facilitated by PT catalysis. Additionally, their EcGUS inhibition activity was evaluated.

## 2. Results

### 2.1. Bioinformatics Analysis and Phylogenetic Tree Construction of PTs

**Figure 2:**
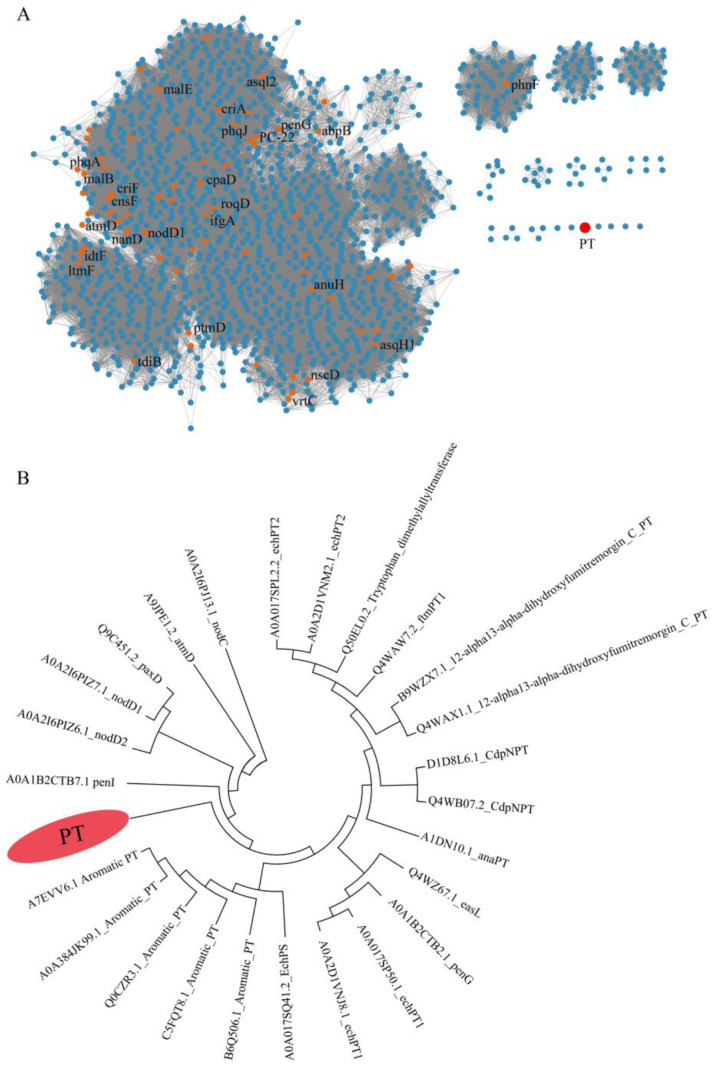
(**A**) Sequence similarity network (SSN) analysis of PTs and their homologous proteins (e-value: 10). (**B**) Phylogenetic tree analysis shows PTs and its homologues.

To explore PTs with different substrates, we conducted a comprehensive analysis of genomic data from our lab, the InterPro database, and the NCBI public database. Nearly 2000 prenyltransferases (PTs), including those from our lab and the InterPro database, were submitted to the Enzyme Function Initiative-Enzyme Similarity Tool (EFI-EST). Subsequently, an SSN of PTs was generated, where enzymes with similar functions clustered together. The SSN results showed that most known proteins clustered together (Figure 2A). A distinct evolutionary clade attracted our attention, as it did not cluster with any specific PTs, suggesting that this PT might be a novel enzyme catalyzing the transfer of prenyl units. In addition, 26 PTs were obtained from our lab and the NCBI public database, and phylogenetic trees were constructed using MEGA 7.0. The phylogenetic tree showed that the 26 PTs were divided into two large branches and five small branches. Among them, a unique small branch attracted our attention, suggesting that this enzyme may have structural novelty and the capability to catalyze the production of isoprenoids (Figure 2B). This unique PT originates from the marine fungus *Penicillium* sp. W21C371, which was sequenced in our laboratory. Comprehensive analysis indicates that this PT possesses distinctive properties, enabling it to catalyze the production of novel compounds. Therefore, further investigation into the secondary metabolites of *Penicillium* sp. W21C371 is warranted.

### 2.2. Structure Elucidation

**Figure 3:**
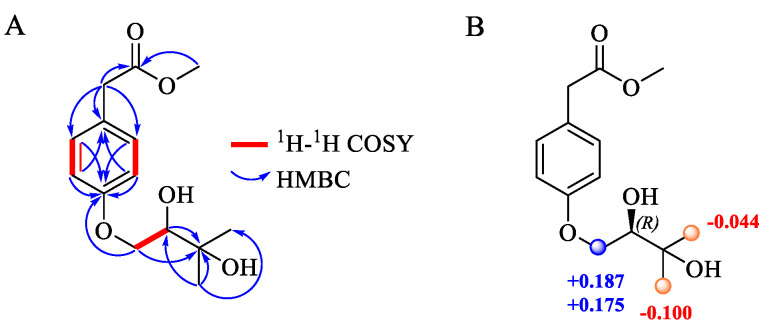
(**A**) Key ^1^H-^1^H COSY and HMBC correlations of (±)-peniprenydiol A (**1**). (**B**) Δ*δ* = *δ*_S_ − *δ*_R_ values 171 in ppm obtained from the MTPA esters of **1b**.

Compound **1** (**1a**/**1b**) was obtained as a colorless oil. The molecular formula of **1** was determined as C_14_H_20_O_5_ by HRESIMS (*m*/*z* 291.1186 [M + Na]^+^, calcd. for C_14_H_20_O_5_Na^+^ 291.1203), indicating five degrees of unsaturation (Figures S14 and S15). The IR spectrum showed characteristic absorption bands for carbonyl (1732 cm^−1^) and hydroxy (3361 cm^−1^) groups (Figure S8). The ^1^H NMR spectrum (Figure S2 and Table 1) displayed proton resonances attributable to two methyls at *δ*_H_ 1.22 (3H, s, H_3_-10) and 1.24 (3H, s, H_3_-11), one methoxy at *δ*_H_ 3.62 (3H, s, H_3_-12), one oxygenated methine at *δ*_H_ 3.75 (dd, *J* = 7.8, 3.0 Hz, H-8), one oxygenated methylene at *δ*_H_ 3.92 (dd, *J* = 9.6, 7.8 Hz, H_b_-7) and 4.26 (dd, *J* = 9.6, 3.0 Hz, H_a_-7), and four aromatic protons at *δ*_H_ 6.90 (2H, d, *J* = 8.4 Hz, H-5 and 5′) and 7.19 (2H, d, *J* = 8.4 Hz, H-4 and 4′). The ^13^C NMR, DEPT, and HSQC spectra (Figures S3–S5) revealed the presence of fourteen carbon resonances, including two methyls at *δ*_C_ 25.6 (C-11) and 26.7 (C-10); one methoxy at *δ*_C_ 51.9 (C-12); two methylenes at *δ*_C_ 40.4 (C-2) and 70.6 (C-7); four aromatic methines at *δ*_C_ 115.4 (C-5), 115.4 (C-5′), 131.1 (C-4), and 131.1 (C-4′); one oxygenated methine at *δ*_C_ 77.1 (C-8); and four non-protonated carbons (one oxygenated at *δ*_C_ 71.9 (C-9), two aromatic at *δ*_C_ 127.4 (C-3) and 159.2 (C-6), and one carbonyl at *δ*_C_ 172.6 (C-1)). The NMR data of **1** were similar to those of westerdijkin A, a hydroxyphenylacetic acid derivative from the deep-sea fungus *Aspergillus westerdijkiae* SCSIO 05233 [23]. Detailed comparative analyses revealed that the signals for the Δ^9^ terminal double bond (*δ*_C_ 143.3 (C-9), *δ*_C_ 112.7 (C-10), *δ*_H_ 5.00 and 5.14 (H_2_-10)) in westerdijkin A were replaced by the newly emerging signals assignable for one methyl (*δ*_H_ 1.22 (H_3_-10) and *δ*_C_ 26.7 (C-10)) and one oxygenated quaternary carbon (*δ*_C_ 71.9 (C-9)) in **1**. The ^1^H-^1^H COSY plots (Figure S6) of H-4/H-5 and H-4′/H-5′, combined with the HMBC (Figure S7) correlations from H-4, H-4′, H-5, and H-5′ to C-6, and from H-5 and H-5′ to C-3, established the *para*-substituted benzene ring moiety in **1**. The HMBC correlations from H_2_-2 to C-1, C-3, C-4, and C-4′, as well as from H_3_-12 to C-1, suggested the presence of a methyl acetate residue that anchored at C-3 via C-2. In addition, the ^1^H-^1^H COSY plot of H_2_-7 (*δ*_H_ 3.92 and 4.26)/H-8 (*δ*_H_ 3.75) and the HMBC correlations from H_2_-7 (*δ*_H_ 3.92 and 4.26) and H-8 (*δ*_H_ 3.75) to the oxygenated quaternary carbon C-9 (*δ*_C_ 71.9) and from the two methyl singlets (*δ*_H_ 1.22 and 1.24) to C-8 (*δ*_C_ 77.1) and C-9 (*δ*_C_ 71.9), in combination with the molecular formula, constructed a 2,3-dihydroxyisopentane moiety that was linked to the benzene ring with an ether linkage between C-6 (*δ*_C_ 159.2) and C-7 (*δ*_C_ 70.6). The planar structure of **1** was thus established (Figure 3).

Compound **1** was initially assumed to be optically pure based on its specific rotation ([α]D20: +14 (c 0.1, MeOH)). Hence, a modified Mosher experiment was performed to determine the absolute configuration of C-8. Unexpectedly, when the (*R*)- and (*S*)-MTPA esters of **1** were subjected to HPLC analysis on a routine ODS C-18 column, two pairs of diastereoisomers were obtained ((*S*)-MTPA**-1a**/(*S*)-MTPA**-1b** and (*R*)-MTPA**-1a**/(*R*)-MTPA**-1b**), suggesting that **1** was probably a partially racemic mixture. This was confirmed by the chiral resolution of **1** on a Chiralpak AD-H column, which afforded a pair of enantiomers **1a** and **1b** in a ratio of ca. 3:7. According to the shielding/deshielding effects of MTPA, the Δ*δ*_H(*S*–_*_R_*_)_ values indicated an 8*R* configuration for (+)**-1b** (Figure 3 and Figures S16 and S17 and Table S2). Thus, **1a** was deduced to be the enantiomer of **1b** based on their identical ^1^H NMR and HRESIMS data (Supplementary Data, Figures S12–S15) and opposite specific rotations ([α]D20: −32 (c 0.05, MeOH) for **1a** and [α]D20: +36 (c 0.1, MeOH) for **1b**). Finally, (−)**-1a** and (+)**-1b** were named (−)-peniprenydiol A and (+)-peniprenydiol A, respectively.

Sixteen known compounds were identified as citridone A (**2**) [24], 5*α*,8*α*-epidioxy-23,24(*R*)-dimethylcholesta-6,9(11),22-trien-3*β*-ol (**3**) [23], 5*α*,8*α*-epidioxy-(23*E*,24*R*)-23-methylergosta-6,22-dien-3*β*-ol (**4**) [25], (22*E*)-23-methylergosta-5,7,22-trien-3*β*-ol (**5**) [26], ergosta-4,6,8(14),22-tetraen-3-one (**6**) [27], isocyathisterol (**7**) [28], ergosterol peroxide (**8**) [29], dankasterone B (**9**) [30], dankasterone (**10**) [31], curvularin (**11**) [32], 10,11-dedrocurvularin (**12**) [33], dehydrocurvularin (**13**) [34], sumalactone A (**14**) [35], morelsin D (**15**) [36], conocenolide A (**16**) [37], and *p*-hydroxy phenylacetic acid (**17**) [38] through the comparison of the spectroscopic data with those reported in the literature. The tremulane sesquiterpenoids, exemplified by morelsin D (**15**) and conocenolide A (**16**) in the present study, were obtained from Ascomycetes for the first time.

### 2.3. Plausible Biosynthetic Pathways of Peniprenydiol A

**Figure 4:**
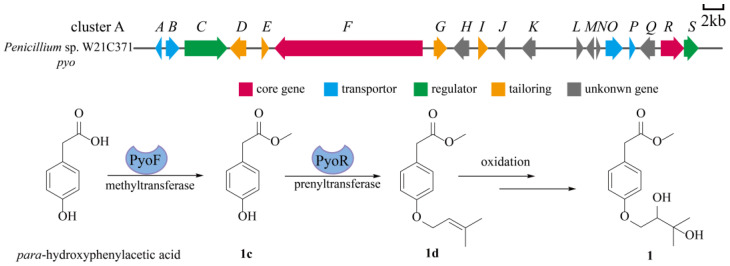
Proposed biosynthetic pathway for **1**.

To investigate the connection between peniprenydiol A and its biosynthetic genes, we obtained the complete genome sequence of the marine fungus *Penicillium* sp. W21C371. The genome was analyzed and predicted using the online tool 2ndfind (https://biosyn.nih.go.jp/2ndfind/, accessed on 1 June 2024). Based on the analysis results, we propose that gene cluster A is responsible for the assembly and transport of peniprenydiol A. This gene cluster comprises multiple genes with diverse functions, including core genes, transporter proteins, regulatory proteins, tailoring enzymes, and genes of unknown function. Using peniprenydiol A as a case study to analyze its biosynthetic pathway, the core PyoF catalyzes the methylation reaction to produce **1c** [39]. This intermediate is then prenylated by the PyoR enzyme to form **1d** [40]. Finally, **1d** is converted into the final product through a non-enzymatic oxidation reaction (Figure 4).

### 2.4. EcGUS Inhibition Assay

**Figure 5:**
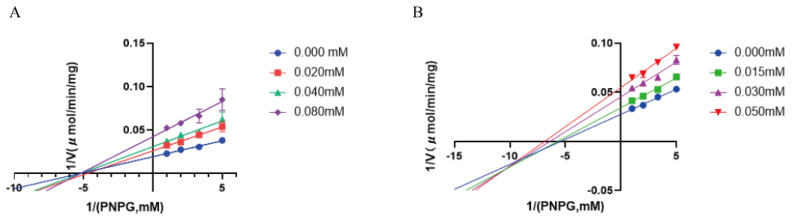
Lineweaver–Burk plots of (**A**) **2**, (**B**) **3** against DSL. All data are expressed as mean ± SD of triplicate reactions.

All isolates **1**–**17** were evaluated for their in vitro inhibitory activity against EcGUS, a potential target for the treatment of drug-induced gastrointestinal disorders, employing D-saccharic acid 1,4-lactone (DSL) as the positive control. In the preliminary screening, compounds **2** and **3** showed relative inhibitory rates of over 60% at a concentration of 100 μM and were subjected to further evaluation. As a result (Table 2), **2** and **3** were found to significantly inhibit the activity of EcGUS in a dose-dependent manner, comparable to that of DSL. In view of the potent inhibitory activity of **2** and **3** against EcGUS, their kinetic mechanisms of inhibition were determined using Lineweaver–Burk plots (Figure 5 and Table S1). The inhibition constants of the enzyme K_i_ and the enzyme–substrate complex K_i_^′^ were obtained by secondary plots of “slope versus [I]” and “Y-intercept versus [I]”, respectively. As shown in Figure 5, the data lines of **2** intersected in the X axis. Meanwhile, the V_max_ value decreased with the increased concentration of **2**, while the K_m_ value did not change. This suggested that **2** was a non-competitive inhibitor of EcGUS that bound with EcGUS and/or the EcGUS–substrate complex at a site other than the active site. The intersection of the data lines of **3** in the third quadrant demonstrated that **3** inhibited EcGUS in a mixed-type manner, which was verified by the changed K_m_ and V_max_ values with the increased concentration of **3**. As a mixed-type inhibitor of EcGUS, **3** was supposed to bind either the free EcGUS or the EcGUS–substrate complex. The inhibition constant K_i_ (89.04 μM) for **3** was larger than K_i_^′^ (48.80 μM), implying that it bound more easily and tightly to the EcGUS–substrate complex than the free EcGUS. To the best of our knowledge, **2** and **3** represent the first citridone and ergosterol derivatives with potent EcGUS-inhibitory activity. However, **2** and **3** were obtained in limited amount in the present study, preventing the in vivo evaluation of their EcGUS inhibition activity. The optimization of the fermentation and separation processes with the aim of increasing the yields of **2** and **3** is underway, which may help to accumulate greater amounts of the two compounds for further in-depth pharmacological studies.

## 3. Materials and Methods

### 3.1. General Experimental Procedures

Optical rotations were obtained with a Rudolph Research Autopol III automatic polarimeter. IR spectra were recorded on a Thermo Nicolet 6700 FT-IR microscope instrument (FT-IR microscope transmission) in KBr pellets. UV spectra were measured on a TU-1900 spectrometer (Persee, Beijing, China). Meanwhile, 1D and 2D-NMR spectra were obtained at 600 MHz for ^1^H and 150 MHz for ^13^C on a Bruker Avance 600 spectrometer, with solvent peaks used as references. HRESIMS data were measured on an Agilent-6210-LC/TOF mass spectrometer (Agilent Technologies, Inc., Santa Clara, CA, USA). EcGUS-inhibitory activity was measured spectrophotometrically using a SpectraMax Plus 384 microplate reader (Molecular Devices, San Jose, CA, USA). Column chromatography (CC) was performed on silica gel (SiO_2_; 200–300 mesh; Qingdao Marine Chemical Co., Ltd.), ODS C-18 gel (50 mm; YMC Co., Ltd., Kyoto, Japan), and MCI CHP20P gel (75–150 μm, Tokyo, Japan). Semi-preparative HPLC was performed on an Cosmosil 5C18-MS-II column (5 μm, 250 × 10 mm) with a Shimadzu LC-20AT system eluting with methanol/water or acetonitrile/water at a flow rate of 3 mL/min. Chiral separation was performed on a Chiralpak AD-H column (5 μm, 4.6 × 250 mm). Thin layer chromatography (TLC) was performed on precoated silica gel GF254 plates (Qingdao Marine Chemical Co., Ltd., Qingdao, China) and visualized by UV light and/or spraying with 10% H_2_SO_4_ in 95% EtOH, followed by heating. All solvents used were of analytical grade and obtained from commercially available sources. All reagents used for biological evaluation were obtained from Sigma-Aldrich (St. Louis, MO, USA).

### 3.2. Fungal Material

The fungus *Penicillium* sp. W21C371 was isolated from a seawater sample collected at Marceau Trench in 2017. It has been deposited in the China Typical Culture Preservation Centre, Wuhan University, Wuhan, China, with the deposit number of CCTCC NO: M2022063. For the identification of the fungus, the genomic DNA was extracted using the Trelief^®^ Hi-Pure Plant Genomic DNA Kit (Tsingke, Beijing, China), according to the manufacturer’s instructions. The primers for the internal transcribed spacer 1 (ITS1) and 4 (ITS4) regions were TCCGTAGGTGAACCTGCGG (5′→3′) and TCCTCCGCTTATTGATATGC (5′→3′), respectively. The PCR amplification of the extracted DNA was performed in a 50 µL reaction mixture consisting of 1 µL gDNA template, 2 µL each of the forward and reverse primers, and 45 µL of Tsingke 1× TSE101 golden mix. The thermocycler was programed with the following PCR conditions: initial denaturation at 98 °C for 2 min, followed by 35 cycles of denaturation at 98 °C for 10 s, annealing at 55 °C for 10 s, and extension at 72 °C for 15 s, with a final extension at 72 °C for 5 min. Upon the completion of amplification, the PCR products were analyzed using gel electrophoresis with a 1% agarose gel (1 g of agarose in 100 mL of Tris buffer) stained with ethidium bromide. Sequencing was carried out by Sanger’s method [41] on a 3730xl DNA Analyzer (Thermo Fisher Scientific, Waltham, MA, USA) at Beijing Tsingke Biotech Co., Ltd. (Beijing, China). The ITS sequence was deposited in GeneBank under Accession No. PP733993.

### 3.3. Bioinformatics Analysis and Phylogenetic Tree Construction of PTs

The sequences of PTs were obtained from our lab, the InterPro database, and the NCBI database. Sequence similarity networks (SSNs) were constructed using the Enzyme Function Initiative (EFI; accessed at https://efi.igb.illinois.edu/, accessed on 1 June 2024) [42]. Cytoscape 3.5.1 was employed for network visualization. Multiple sequence alignments were performed using MUSCLE. Phylogenetic trees were constructed using the maximum likelihood method in MEGA6 and visualized using the Interactive Tree of Life (ITOL, http://itol.embl.de/, accessed on 1 June 2024).

### 3.4. Fermentation and Extraction

The fungus was cultured on potato dextrose agar (PDA) plates for 5 days at 28 °C. The spores were washed with 10 mL sterile water containing 2% tween 80 and aseptically inoculated into 45 conical flasks (1000 mL) containing rice medium (rice 100 g, distilled water 135 mL, sterilized at 121 °C for 20 min and cooled). The conical flasks were kept at 28 °C. After culturing for 20 days, the collected cultures were mashed and soaked in 95% ethanol for 4 days at room temperature and filtered (repeated 3 times). The filtrate was condensed in a vacuum to give a crude extract (550 g), which was suspended in distilled water (1.5 L) and partitioned with EtOAc (1.5 L) 3 times. The EtOAc layer was combined and concentrated in a vacuum to yield an EtOAc-soluble extract (64 g).

### 3.5. Isolation and Purification

The EtOAc-soluble extract (64 g) was subjected to MCI CHP20P CC eluting with a gradient of MeOH-H_2_O (20:80→100:0, *v*/*v*) to offer six fractions (Fr. A–F). Fr. B (2.26 g) was fractionated by silica gel CC eluting with a gradient of CH_2_Cl_2_-MeOH (60:1→40:1, *v*/*v*) to give five sub-fractions (Fr. B1–B5). Fr. B1 was separated by semi-preparative HPLC eluting with MeOH/H_2_O (35:65, *v*/*v*) to give **1** (21.1 mg, t_R_ = 10.5 min). Fr. B2 was subjected to semi-preparative HPLC eluting with CH_3_CN-H_2_O (45:55, *v*/*v*) to afford **13** (1 mg, t_R_ = 11.5 min). Fr. C (8.56 g) was subjected to silica gel CC eluting with a gradient of CH_2_Cl_2_-MeOH (40:1→5:1, *v*/*v*) to give three sub-fractions (Fr. C1–C3). Fr. C2 was separated by semi-preparative HPLC eluting with MeOH/H_2_O (40:60, *v*/*v*) to yield **14** (2 mg, t_R_ = 16.5 min) and **17** (6.5 mg, t_R_ = 14.5 min). Fr. D (14.7 g) was subjected to silica gel CC eluting with a gradient of CH_2_Cl_2_-MeOH (50:1→30:1, *v*/*v*) to give **11** (1.5 g) and four sub-fractions (Fr. D1–D4). Fr. D1 was chromatographed over ODS C-18 gel eluting with a gradient of MeOH-H_2_O (60:40→80:20, *v*/*v*) to furnish **12** (37.7 mg), **15** (19.5 mg), and **16** (5.4 g). The purification of Fr. E (12.5 g) by silica gel CC eluting with a gradient of CH_2_Cl_2_-MeOH (50:1→20:1, *v*/*v*) yielded four sub-fractions (Fr. E1-Fr. E4). Fr. E2 was subjected to silica gel CC eluting with a gradient of MeOH-H_2_O (80:20→90:10, *v*/*v*) to afford **9** (17 mg). Moreover, **2** (15.4 mg) was obtained from Fr. E4 by silica gel CC eluting with a gradient of MeOH-H_2_O (80:20→90:10, *v*/*v*). Fr. F (12.5 g) was first separated on a silica gel column eluting with a gradient of CH_2_Cl_2_-MeOH (50:1→20:1, *v*/*v*) to yield four sub-fractions (Fr. F1–F4). Fr. F1 was subjected to ODS C-18 CC and eluted with a gradient of MeOH-H_2_O (75:25→100:0, *v*/*v*) to offer **6** (10 mg). Fr. F2 was separated on an ODS C-18 column eluting with a gradient of MeOH-H_2_O (80:20→100:0, *v*/*v*) to furnish **5** (55.0 mg), **4** (4 mg), and **7** (15.3 mg). Fr. F3 was purified by semi-preparative HPLC eluting with MeOH-H_2_O (95:5, *v*/*v*) to give **10** (3 mg, t_R_ = 15.5 min). Fr. F4 was separated by semi-preparative HPLC eluting with MeOH/H_2_O (95:5, *v*/*v*) to furnish **3** (1.7 mg, t_R_ = 10.0 min) and **8** (15.2 mg, t_R_ = 12.0 min). Finally, **1** (5.0 mg) was separated by chiral HPLC eluting with isopropanol/n-hexane (18:82, *v*/*v*) at a flow rate of 1 mL/min to yield **1a** (0.8 mg, t_R_ = 16.7 min) and **1b** (3.2 mg, t_R_ = 22.0 min) (Figure S11).

### 3.6. Spectroscopic Data

Peniprenydiol A (**1**), colorless oil, [α]D20: +14 (c 0.1, MeOH), IR (KBr) (Figure S8): 3361, 2921, 2850, 1732, 1659, 1632, 1612, 1584, 1513, 1462, 1435, 1297, 1245, 1157, 1094, 1030, 805 cm^−1^. UV λ_max_ (MeOH) nm (log ɛ): 207 (3.91) (Figure S10); HR-ESI-MS *m*/*z*: 291.1186 [M + Na]^+^ (calcd. for C_14_H_20_O_5_Na^+^, 291.1203). For ^1^H- and ^13^C-NMR data, see Table 1.

(−)-Peniprenydiol A (**1a**), colorless oil, [α]D20: −32 (c 0.05, MeOH), HR-ESI-MS *m*/*z* 291.1202 [M + Na]^+^ (calcd. for C_14_H_20_O_5_Na^+^, 291.1203). For ^1^H-NMR data, see Figure S12. 

(+)-Peniprenydiol A (**1b**), colorless oil, [α]D20: +36 (c 0.1, MeOH), HR-ESI-MS *m*/*z* 291.1202 [M + Na]^+^ (calcd. for C_14_H_20_O_5_Na^+^, 291.1203). For ^1^H-NMR data, see Figure S13.

### 3.7. Preparation of (R)- and (S)-MTPA Esters of **1b**

Compound **1** (1 mg, 3.73 μmol) was dissolved in 0.5 mL of anhydrous pyridine-d_5_, and (*S*)-MTPA chloride (5 μL, 26.5 μmol) was added under N_2_. The mixture was allowed to react for 36 h at room temperature. Further purification of the (*R*)-MTPA ester of **1** using semi-preparative HPLC with an ODS C18 column afforded the (*R*)-MTPA esters of **1a** and **1b**. The (*S*)-MTPA esters of **1a** and **1b** were prepared with (*R*)-MTPA chloride and were purified in the same manner [43].

### 3.8. Bioassay

The EcGUS inhibition assay was performed according to the method previously reported, employing DSL as the positive control [44]. The results are presented as means ± SD for three independent experiments.

## 4. Discussion

With the rapid development of bioinformatics, genome mining strategies have been widely applied to discover new natural products. By combining SSN analysis and phylogenetic tree construction, we can identify enzymes and genes with potential new functions. Using this approach, we discovered a unique PT that can transfer an isopropyl group to an aromatic nucleus, forming C-C or C-O bonds. Utilizing PT-guided separation techniques, we isolated a pair of novel hydroxyphenylacetic acid derivative enantiomers with prenyl units, (±)-peniprenydiol A, from the rice extracts of *Penicillium* sp. W21C371. The structure was formed through a crucial step involving PT catalysis, based on the known structure of p-hydroxy phenylacetic acid. In natural product studies, these compounds can appear in different enantiomeric forms. One form is a racemate, an equal mixture of two enantiomers, while the other form consists of non-racemic enantiomers in an unequal mixture. Currently, researchers focus more on racemic natural products, while non-racemic enantiomers are largely overlooked. This is because the optical purity of new natural products is rarely reported upon publication. It is usually assumed that when new chiral natural products exhibit optical activity, they are also optically pure. We measured the optical rotation of **1** [α]D20: +14 (c 0.1, MeOH) and considered it optically pure. Subsequently, using a modified Mosher method to determine the configuration of its 8-OH group, we found that the compound exists as non-racemic enantiomers in unequal proportions. This compound was separated by chiral chromatography to obtain a pair of non-racemic enantiomers, (−)-**1** and (+)-**1**.

Citridone A (**2**), featuring a unique phenyl-*R*-furopyridone skeleton (6-6/5/5 ring system), belongs to the citridone family of fungal pyridines and was first isolated by Ōmura and co-workers from *Penicillium* sp. FKI-1938. It was identified as a potentiator of miconazole against *Candida albicans* [45]. Additionally, it could inhibit the biosynthesis of staphyloxanthin, a key virulence factor in methicillin-resistant *Staphylococcus aureus* (MRSA), rendering it a promising antibiotic lead with a new mode of action [46]. As a result, citridone A (**2**) has drawn attention from both chemists and biochemists. The total synthesis of citridone A (**2**) was accomplished in 2011 [47], while the biosynthetic pathway was unveiled in 2020 [48]. Compounds **3**–**10** are derivatives of ergosterol, a representative fungisterol that is a component of the fungal cell membrane and determines the fluidity, permeability, and activity of membrane-associated proteins in fungi [49]. Compounds **3**–**5** are notable in this series of fungal sterols for harboring an additional C-23 methyl group, which was proposed to be formed by *S*-adenosyl-*L*-methine (SAM) [50]. Preliminary structure–activity relationship studies suggest that both the C-23 methyl group and Δ^9(11)^ double bond play important roles in retaining the EcGUS-inhibitory activity, as seen in the activity of **3**, **4**, and **8**. To the best of our knowledge, **2** and **3** represent the first citridone and ergosterol derivatives with potent EcGUS-inhibitory activity. Morelsin D (**15**) and conocenolide A (**16**) are tremulane sesquiterpenoids that have been only reported from Basidiomycetes so far. It is worth noting that our work represents the first report of tremulane sesquiterpenoids from Ascomycetes. The well-established genetic manipulation systems in Ascomycetes, particularly within the genus *Penicillium*, may facilitate biosynthetic studies and the metabolic engineering of this type of sesquiterpenoid. 

## 5. Conclusions

In summary, we conducted a comprehensive genome mining analysis using genomic data from our lab, the InterPro database, and the publicly available NCBI database. Through this analysis, we identified a unique PT capable of catalyzing the transfer of a prenyl group to an aromatic nucleus, forming C-C or C-O bonds. From the marine fungus *Penicillium* sp. W21C371, a pair of novel hydroxyphenylacetic acid derivative enantiomers, (±)-peniprenydiol A (**1**) with prenyl units, along with sixteen known compounds **2**–**17**, were isolated through PT-guided isolation. The plausible biosynthetic pathways of compound **1** were deduced. This study also marks the first time that tremulane sesquiterpenoids, exemplified by morelsin D (**15**) and conocenolide A (**16**), have been obtained from Ascomycetes. Additionally, compounds **2** and **3** were identified as potent EcGUS inhibitors for the first time, exhibiting non-competitive and mixed-type inhibition, respectively.

## Figures and Tables

**Table 1:** ^1^H (600 MHz) and ^13^C NMR (150 MHz) data of **1** in Acetone-*d*_6_ (*δ*_H_ in ppm, *J* in Hz).

Position	*δ*_C_, Type	*δ*_H_
1	172.6, C	-
2	40.4, CH_2_	3.56, s
3	127.4, C	-
4	131.1, CH	7.19, d (8.4)
4′	131.1, CH	7.19, d (8.4)
5	115.4, CH	6.90, d (8.4)
5′	115.4, CH	6.90, d (8.4)
6	159.2, C	-
7	70.6, CH_2_	3.92, dd, (9.6, 7.8)
		4.26, dd, (9.6, 3.0)
8	77.1, CH	3.75, dd (7.8, 3.0)
9	71.9, C	-
10	26.7, CH_3_	1.22, s
11	25.6, CH_3_	1.24, s
12	51.9, CH_3_	3.62, s

**Table 2:** Inhibitory activity of **2** and **3** against EcUGS.

Compound	IC_50_ (μM)
**2**	44.60 ± 0.84
**3**	21.60 ± 0.76
DSL	47.94 ± 0.89

## Data Availability

Data are contained within the article or Supplementary Materials.

## Supplementary Materials

The following supporting information can be downloaded at: https://www.mdpi.com/article/10.3390/md22070296/s1, Table S1: Inhibition kinetics of **2** and **3** against EcGUS; Table S2: Selected ^1^H (600 MHz) NMR data of **1b** and its MTPA esters in Pyridine-*d*_5_ (*δ*_H_ in ppm, *J* in Hz); Table S3: Putative functions of selected genes from peniprenydiol A gene cluster. Figure S1: HPLC-DAD chromatogram of twenty-one extracts of *Penicillium* sp. W21C371; Figure S2: ^1^H NMR spectrum of **1** in Acetone-*d*_6_ (600 MHz); Figure S3: ^13^C NMR spectrum of **1** in Acetone-*d*_6_ (150 MHz); Figure S4: DEPT spectrum of **1** in Acetone-*d*_6_; Figure S5: HSQC spectrum of **1** in Acetone-*d*_6_; Figure S6: ^1^H-^1^H COSY spectrum of **1** in Acetone-*d*_6_; Figure S7: HMBC spectrum of **1** in Acetone-*d*_6_; Figure S8: The IR spectrum of **1**; Figure S9: The HRESIMS spectroscopic data of **1**; Figure S10: UV spectrum of **1**; Figure S11: Chiral HPLC separation profile of **1a**/**1b;** Figure S12: ^1^H NMR spectrum of **1a** in Pyridine-*d*_5_ (600 MHz); Figure S13: ^1^H NMR spectrum of **1b** in Pyridine-*d*_5_ (600 MHz); Figure S14: The HRESIMS spectrum of compound **1a;** Figure S15: The HRESIMS spectrum of compound **1b;** Figure S16: ^1^H NMR spectrum of (*R*)-MTPA ester of **1b** in Pyridine-*d*_5_ (600 MHz); Figure S17: ^1^H NMR spectrum of (*S*)-MTPA ester of **1b** in Pyridine-*d*_5_ (600 MHz).
