# Supplementary material for: Discovery of Prenyltransferase-Guided Hydroxyphenylacetic Acid Derivatives from Marine Fungus Penicillium sp. W21C371
Source: Mar Drugs. 2024 Jun 26;22(7):296. doi: 10.3390/md22070296 (PMC11278016; doi:10.3390/md22070296)
Supplement: Supplementary file 1 [file marinedrugs-22-00296-s001.zip › marinedrugs-3071872-supplementary.pdf]

Supplementary Materials

Discovery of Prenyltransferase-Guided Hydroxyphenylacetic Acid Derivatives from Marine Fungus *Penicillium* sp. W21C371

Cancan Wang [1,†], Ye Fan [1,†], Chenjie Wang [1], Jing Tang [1], Yixian Qiu [1], Keren Xu [1], Yingjia Ding [1], Ying Liu [1], Youmin Ying [1,*] and Hong Wang [1,*]

[1] College of Pharmaceutical Science & Collaborative Innovation Center of Yangtze River Delta Region Green Pharmaceuticals, Zhejiang University of Technology, Hangzhou 310014, China; cancanw1120@163.com (C.W.); m18767175381_1@163.com (Y.F.); 211123070063@zjut.edu.cn (C.W.); t_joyce@126.com (J.T.); 13588779931@163.com (Y.Q.); 201805150118@zjut.edu.cn (K.X.); 19857445690@163.com (Y.D.); 15844357993@163.com (Y.L.)

* Correspondence: ymying@zjut.edu.cn (Y.Y.); hongw@zjut.edu.cn (H.W.)

† These authors contributed equally to this work.

Contents

Table S1. Inhibition Kinetics of **2** and **3** against EcGUS. 3

Table S2. Selected ^{1}H (600 MHz) NMR Data of **1b** and its MTPA esters in Pyridine-d_5 (δ_H in ppm, J in Hz). 3

Table S3. Putative functions of the Selected genes from peniprenydiol A gene cluster. 3

Figure S1. HPLC-DAD chromatogram of twenty-one extracts of *Penicillium* sp. W21C371. 4

Figure S2. ^{1}H NMR spectrum of **1** in Acetone-d_6 (600 MHz). 5

Figure S3. ^{13}C NMR spectrum of **1** in Acetone-d_6 (150 MHz). 5

Figure S4. DEPT spectrum of **1** in Acetone-d_6. 6

Figure S5. HSQC spectrum of **1** in Acetone-d_6. 6

Figure S6. ^{1}H-^{1}H COSY spectrum of **1** in Acetone-d_6. 7

Figure S7. HMBC spectrum of **1** in Acetone-d_6. 7

Figure S8. The IR spectrum of **1**. 8

Figure S9. The HRESIMS spectroscopic data of **1**. 8

Figure S10. UV spectrum of **1**. 8

Figure S11. Chiral HPLC separation profile of **1a**/**1b**. 9

Figure S12. ^{1}H NMR spectrum of **1a** in Pyridine-d_5 (600 MHz). 9

Figure S13. ^{1}H NMR spectrum of **1b** in Pyridine-d_5 (600 MHz). 10

Figure S14. The HRESIMS spectrum of compound **1a**. 10

Figure S15. The HRESIMS spectrum of compound **1b**. 10

Figure S16. ^{1}H NMR spectrum of (*R*)-MTPA ester of **1b** in Pyridine-d_5 (600 MHz). ... 11

Figure S17. ^{1}H NMR spectrum of (*S*)-MTPA ester of **1b** in Pyridine-d_5 (600 MHz). ... 11

Table S1. Inhibition Kinetics of **2** and **3** against EcGUS.

Compounds	Concentration (mM)	K_m (mM)	V_{max} (mg/μmol·min)	K_i (μM)	K_i' (μM)
**2**	0.00	0.19 ± 0.03	51.86 ± 2.54	76.34	69.20
	0.02	0.21 ± 0.04	38.33 ± 2.97		
	0.04	0.19 ± 0.05	32.36 ± 3.11		
	0.08	0.19 ± 0.05	23.58 ± 2.49		
**3**	0.000	0.18 ± 0.02	36.11 ± 1.35	89.04	48.80
	0.015	0.17 ± 0.02	29.05 ± 1.14		
	0.030	0.16 ± 0.02	22.03 ± 1.06		
	0.050	0.15 ± 0.02	18.22 ± 0.64		

Table S2. Selected ^{1}H (600 MHz) NMR Data of **1b** and its MTPA esters in Pyridine-d_5 (δ_H in ppm, J in Hz).

Position	δ_H		
	**1b**	**(*R*)-MTPA ester of 1b**	**(*S*)-MTPA ester of 1b**
7	4.72, dd (9.6, 2.3)	4.655, brd (10.5)	4.842, brd (10.5)
	4.39, dd (9.6, 8.1)	4.326, dd (9.6, 9.6)	4.501, dd (9.6, 9.6)
8	4.31, dd (8.1, 2.3)	5.99, brd (8.0)	5.99, brd (8.0)
10	1.59, s	1.522, s	1.478, s
11	1.62, s	1.527, s	1.427, s

Table S3. Putative functions of the selected genes from peniprenydiol A gene cluster.

gene	left	right	domain (Pfam)	homology (swissprot)
29	80310	95295	Methyltransferse	P9WET4.1
41	119369	121733	Prenyltransferase	Q4WES9.1

AA sequence of prenyltransferase:

MTSHIAPWRTSAQGHLTPDENGDLKTDYSRWRLVNEEGRQTWRYLESDEENSAWLQTVA

EKYHLGLPTGLPILPEAKTPLQAAENGLSFFSHLQLDAGNWACEYGGPMFMIPSIIVAYYVT

NTPIPAAYATEIKRYLFARQHKEDGGWGLHIEGHSSVFGTAMNYVVLRIIGVHEDDPRMIK

ARGLLHKFGGAIYGPHWAKAFLSILGVMKWDAVNPVPPEIWMLPDWVPFAPWRWWIHI

RQVFLPLSYIWSKKWSMPLNDLTKQLREEIYAQPYESIDFASHRNSIHDADNYYPKTWVLN

SINEILVRLYNPLFRIGPVVRRSEEWVWDLICMEDQNTDYAGVGPVSNPLNMICCYIHDGP

GSETVRRHEYRLNDYMWVKHEGMLANGTNGVQVWDTTFATQAVVIAGLADDPKWRPM

LSRALEFIEDHQLRENVPRQEECYRQHRKGAWPFSTKVQGYTVSDCTAEGLRSTLQLQEM

HGFPKLISLDRLKDSVDCLLLLQNPSGGFSEYESTRASPKIECLNAAEVFGGIMISYDHPECT

TASVTALSLFSKFYPDYRADEIRAAKEKAVKCIKRMQRDDGSWYGSWGICFTYAAMFALES

LASIGETYETSENSHRGCEFLISKQQADGGWGESYLSSEKHVYVQHEMSQVTQTAWACLA

LMEAEYPHKEPLEKAMKLLMSRQQANGEWLQESIEGVFNQSCMISYPNYKFYWPIRALGL

YSRKFGNAELK

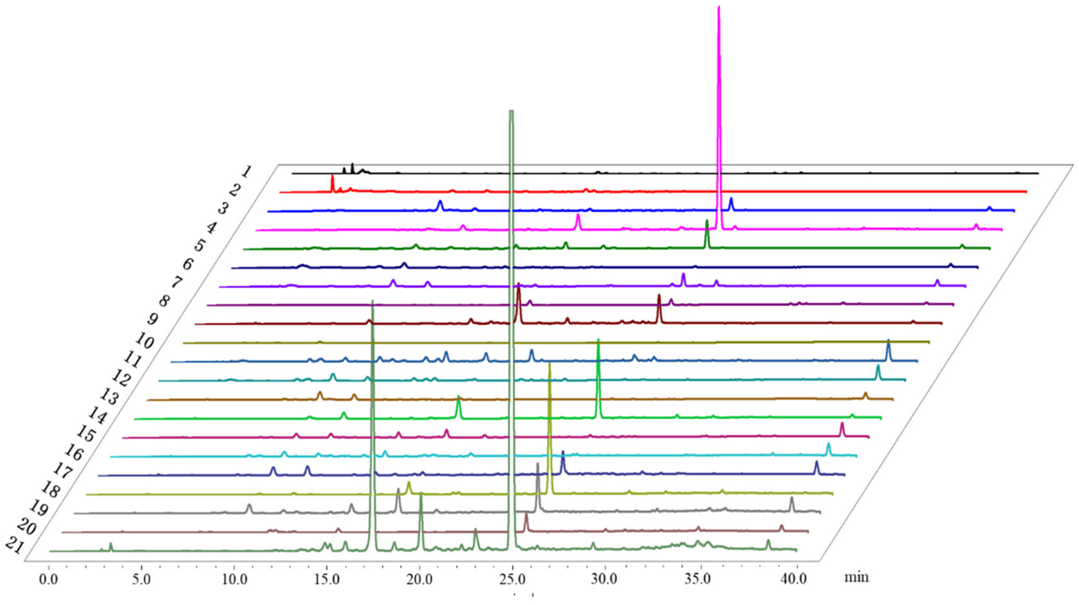

Figure S1. HPLC-DAD chromatogram of twenty-one extracts of *Penicillium* sp. W21C371.

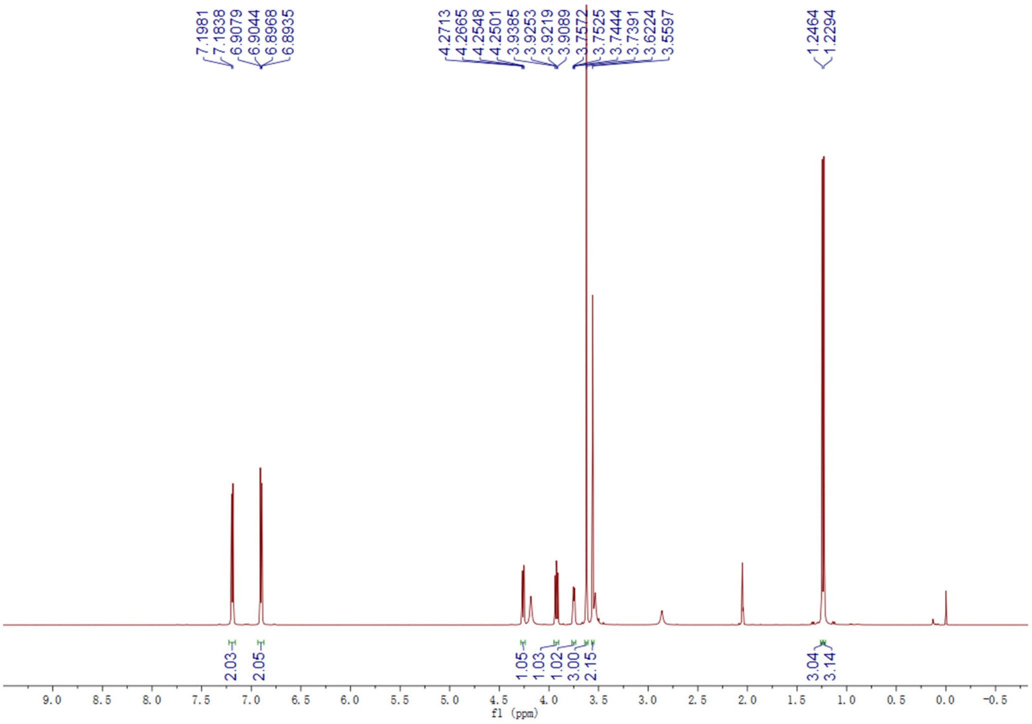

Figure S2. ^{1}H NMR spectrum of **1** in Acetone-d_6 (600 MHz).

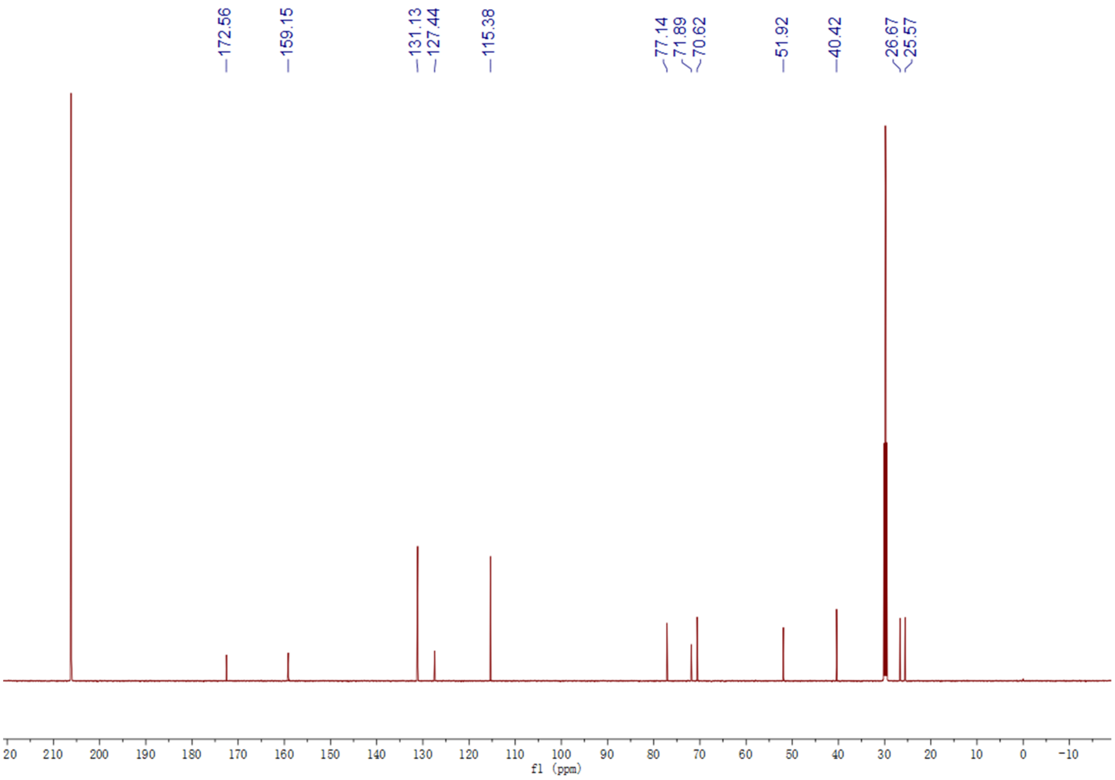

Figure S3. ^{13}C NMR spectrum of **1** in Acetone-d_6 (150 MHz).

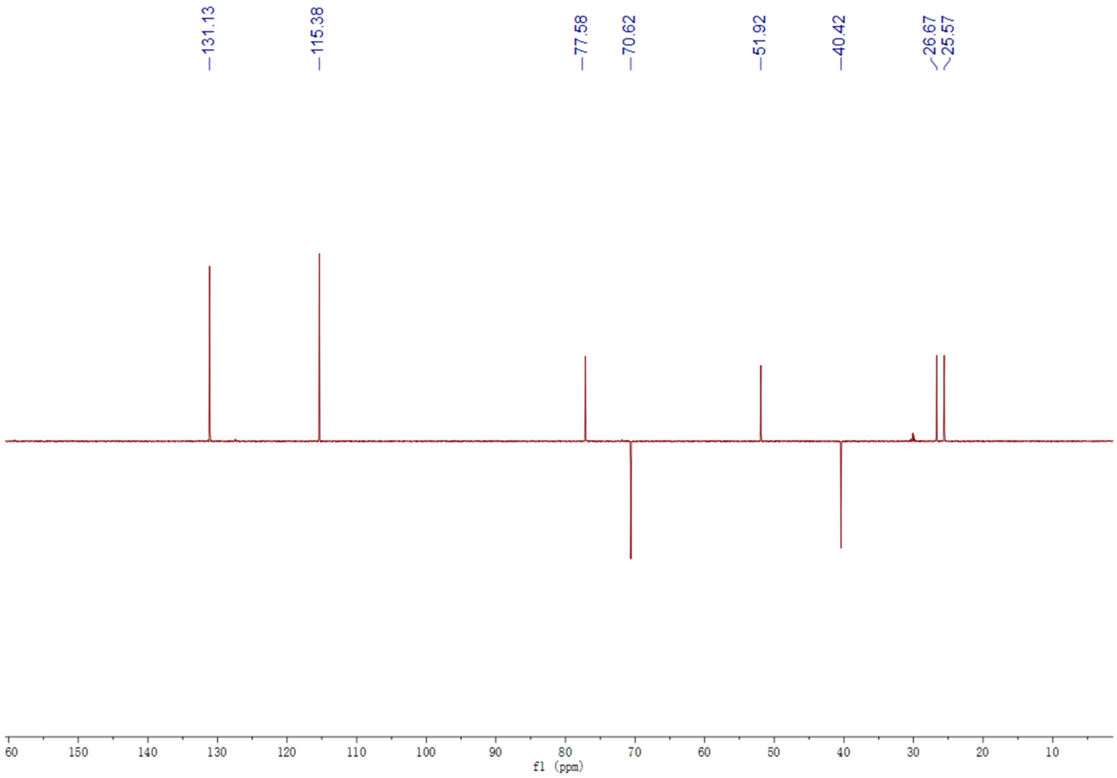

Figure S4. DEPT spectrum of **1** in Acetone-d_6.

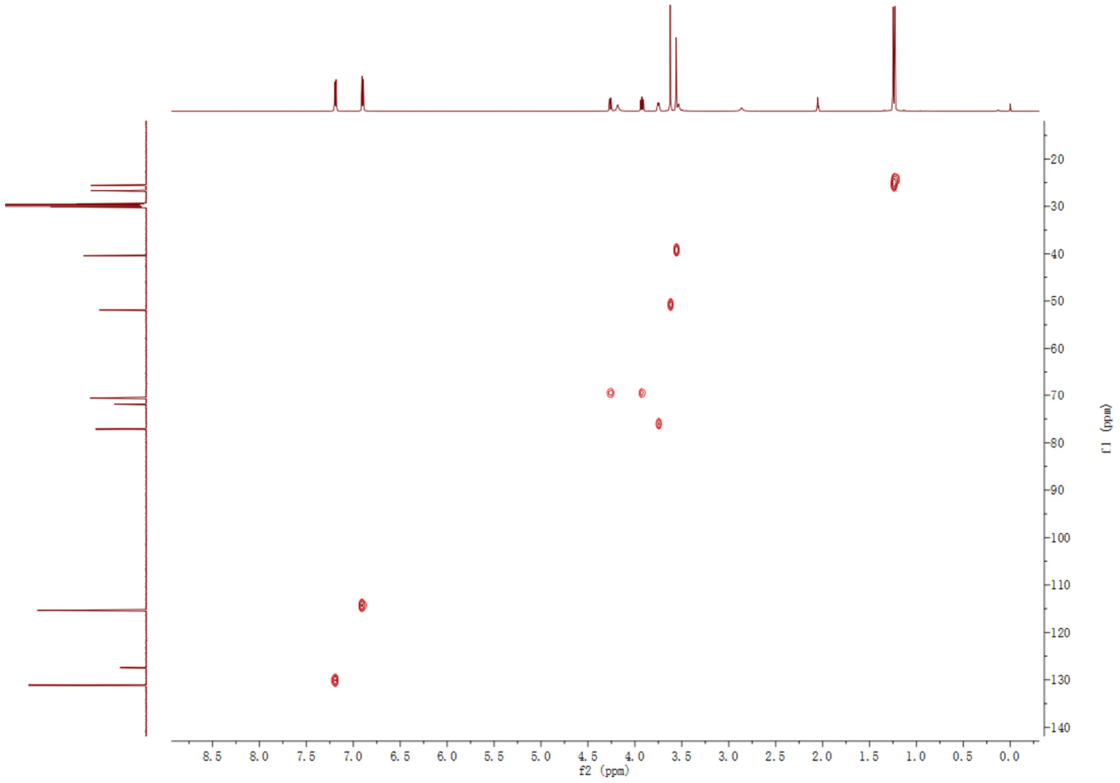

Figure S5. HSQC spectrum of **1** in Acetone-d_6.

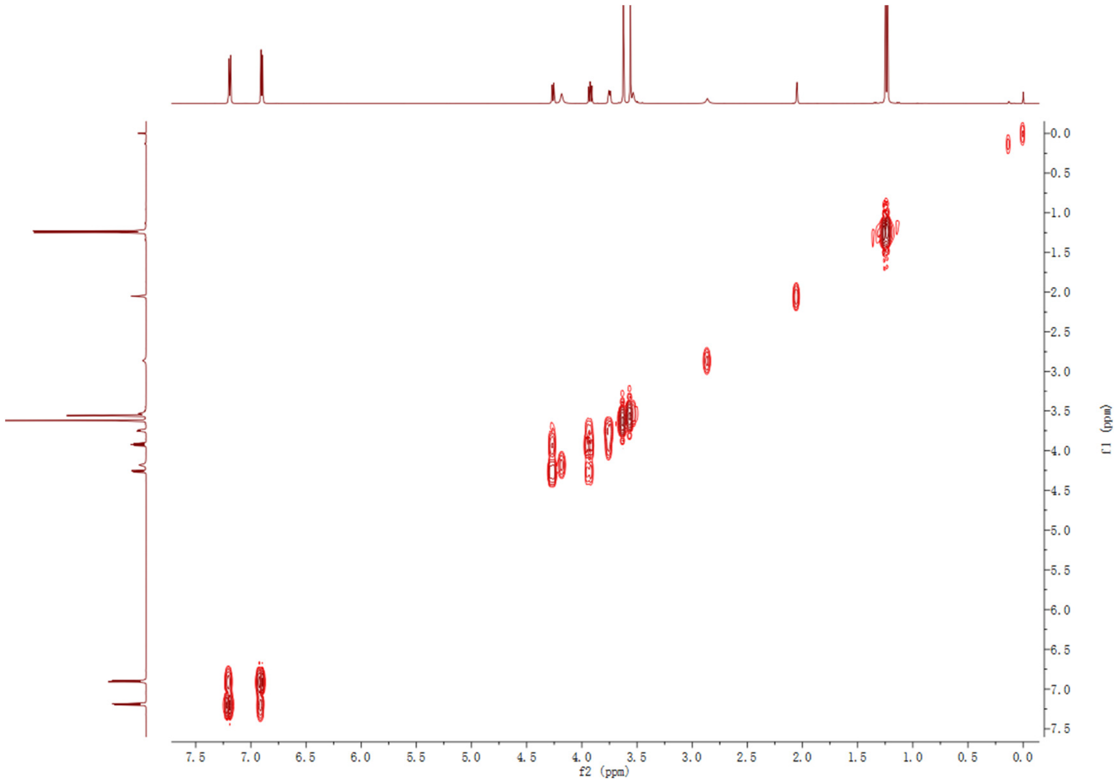

Figure S6. ^{1}H-^{1}H COSY spectrum of **1** in Acetone-d_6.

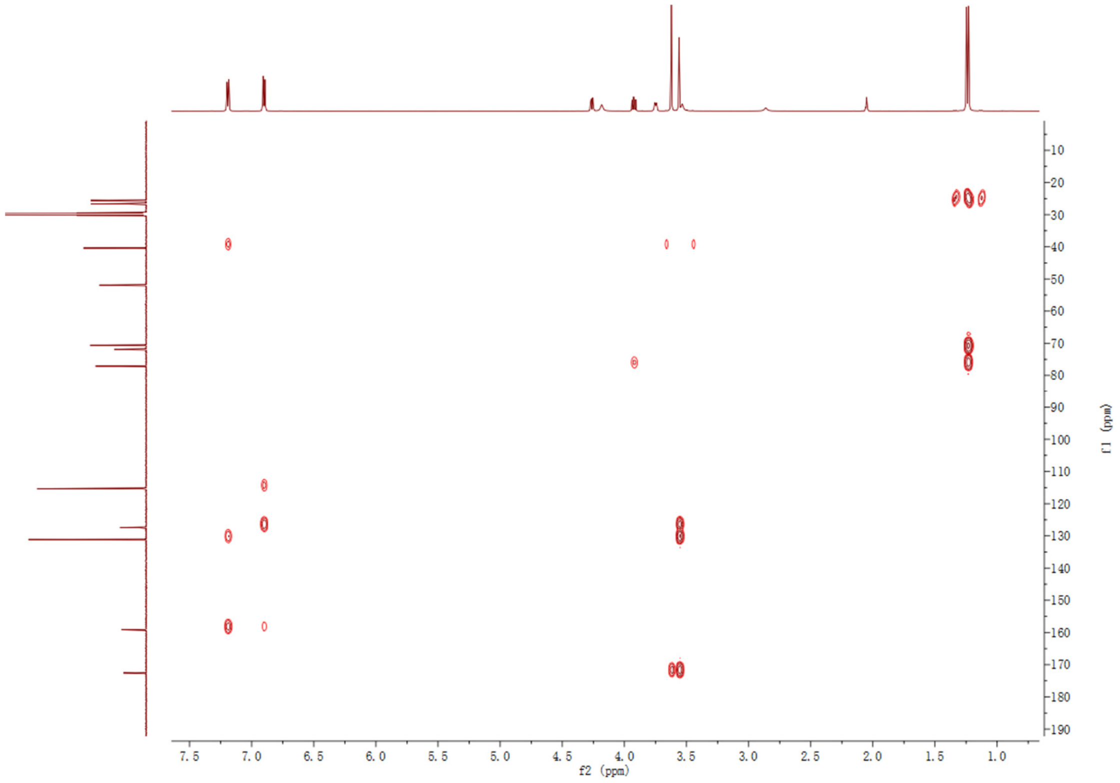

Figure S7. HMBC spectrum of **1** in Acetone-d_6.

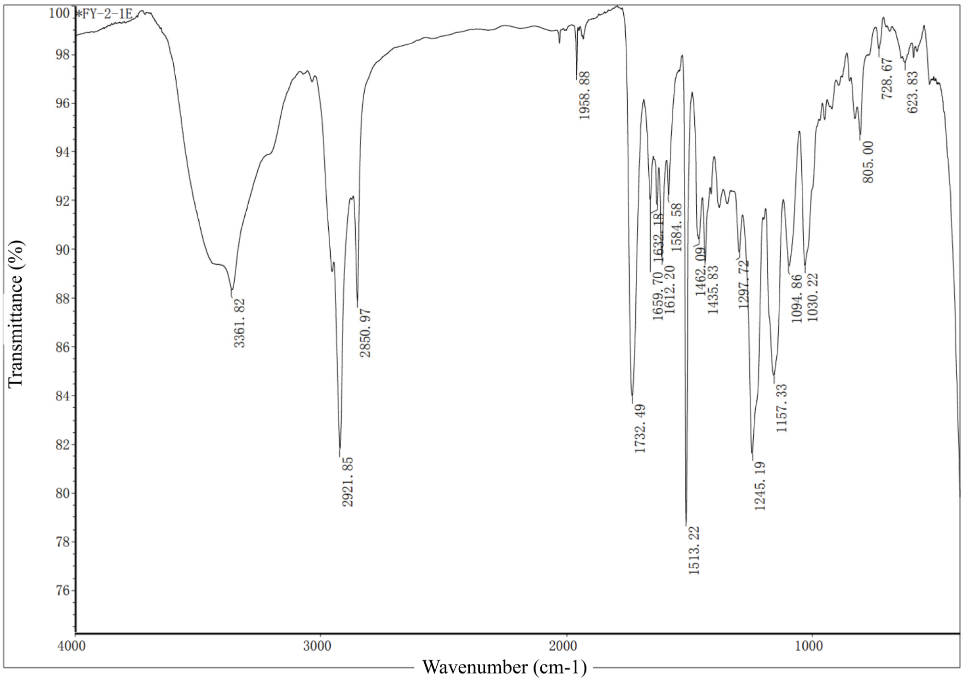

Figure S8. The IR spectrum of **1**.

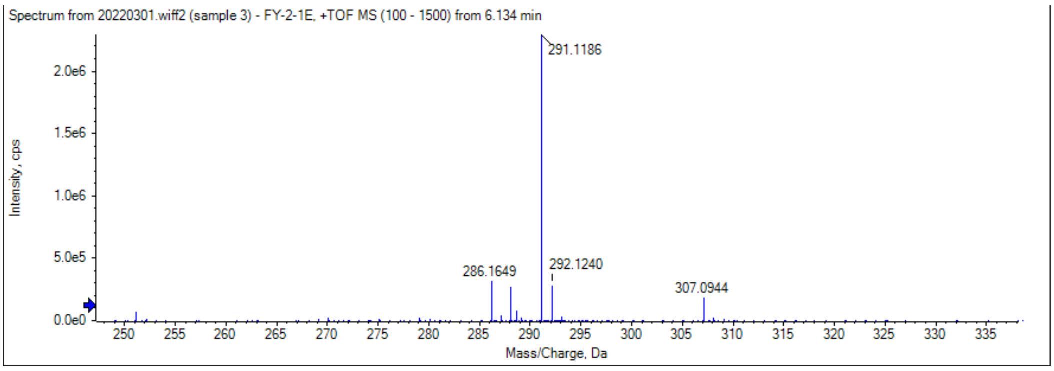

Figure S9. The HRESIMS spectroscopic data of **1**.

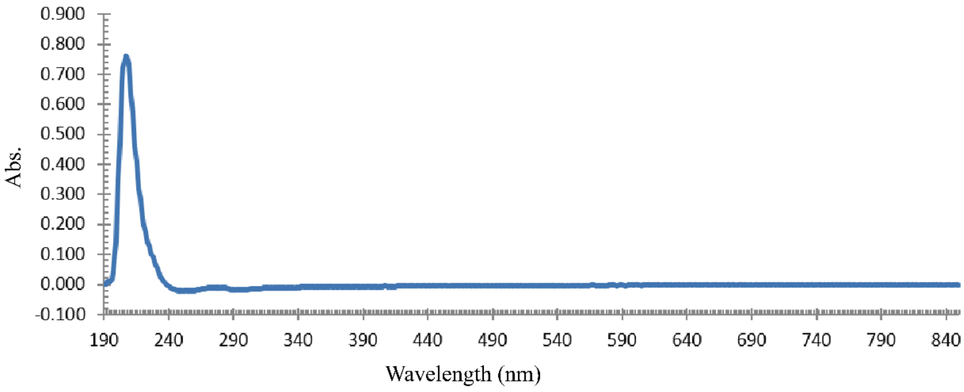

Figure S10. UV spectrum of **1**.

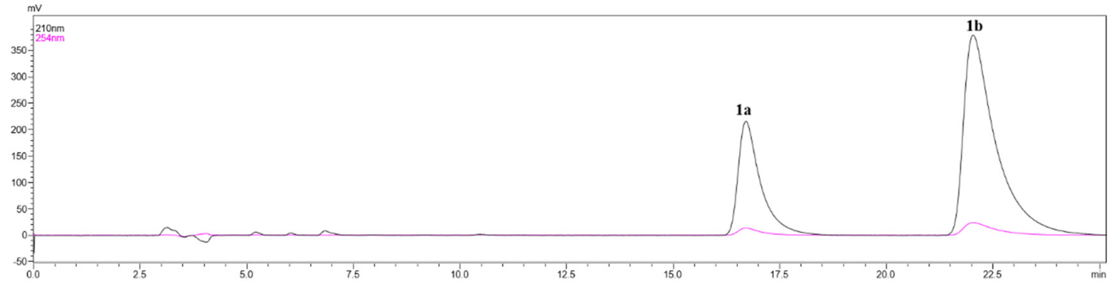

Figure S11. Chiral HPLC separation profile of **1a**/**1b**.

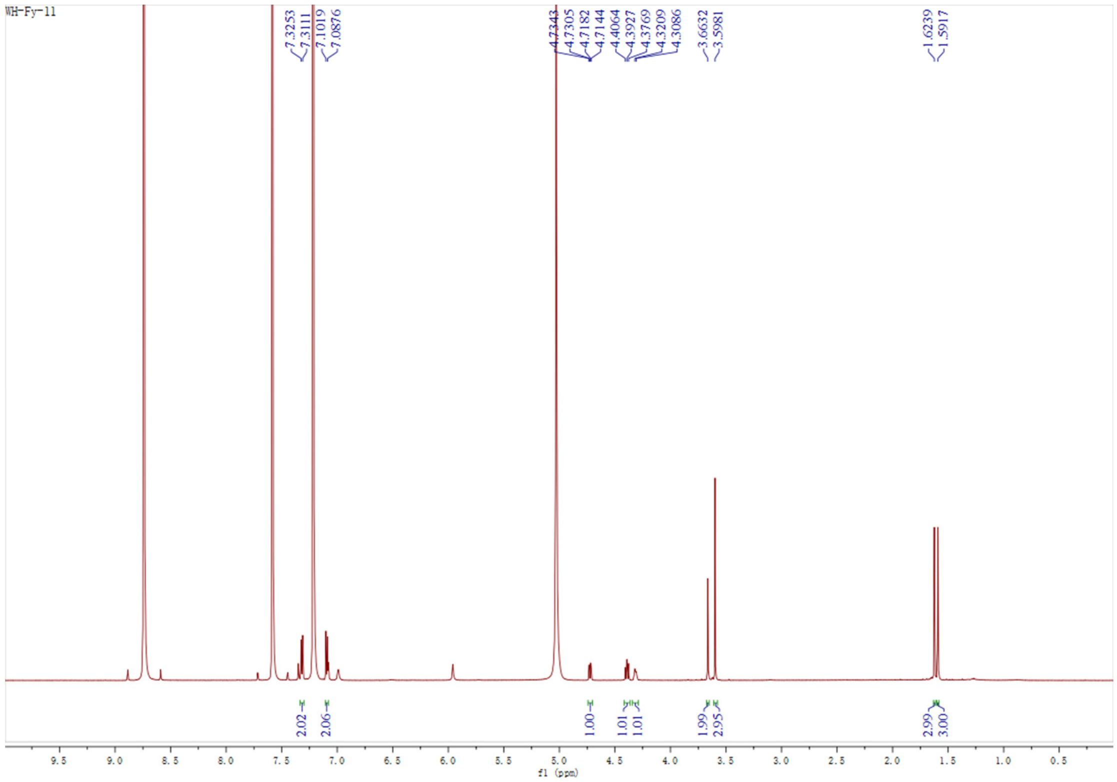

Figure S12. ^{1}H NMR spectrum of **1a** in Pyridine-d_5 (600 MHz).

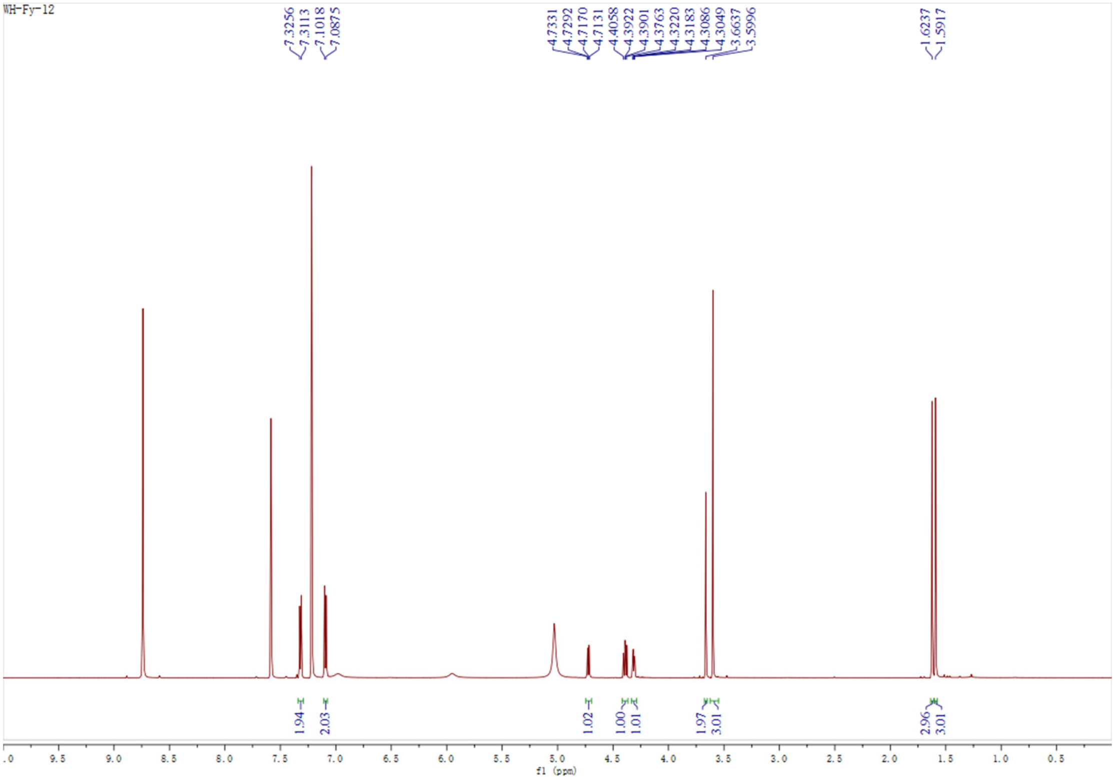

Figure S13. ^{1}H NMR spectrum of **1b** in Pyridine-*d*5 (600 MHz).

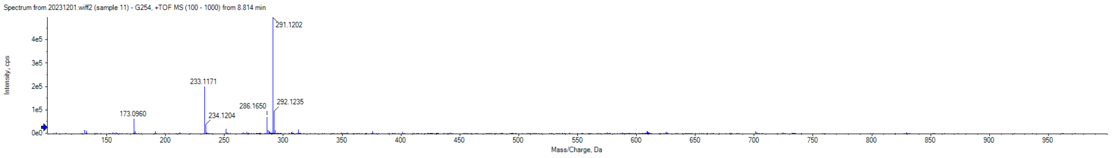

Figure S14. The HRESIMS spectrum of compound **1a**.

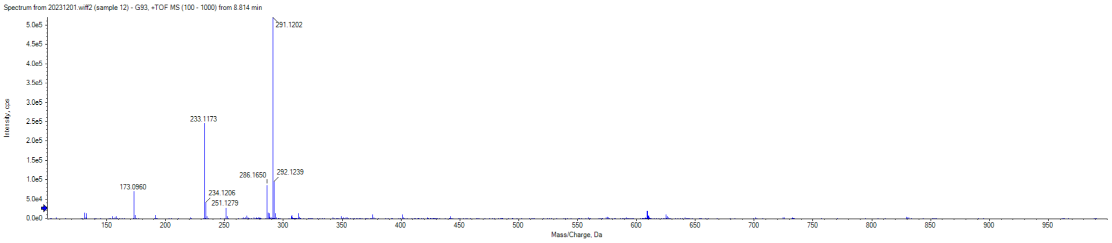

Figure S15. The HRESIMS spectrum of compound **1b**.

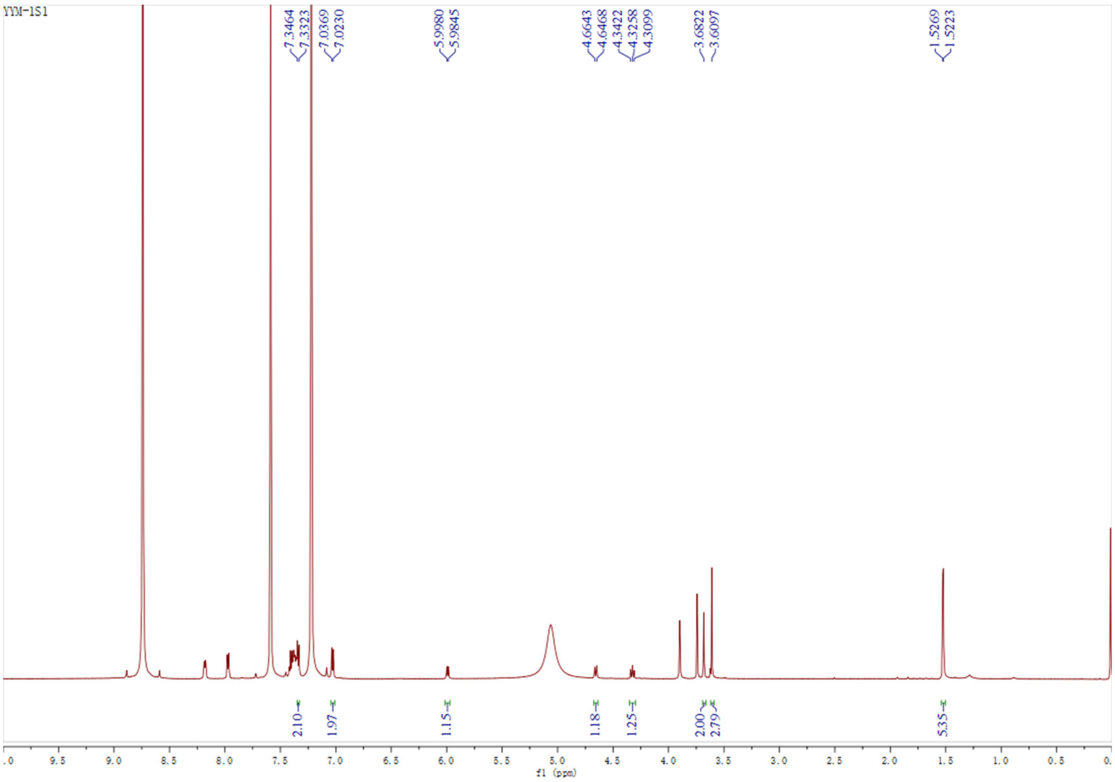

Figure S16. ^{1}H NMR spectrum of (*R*)-MTPA ester of **1b** in Pyridine-d_5 (600 MHz).

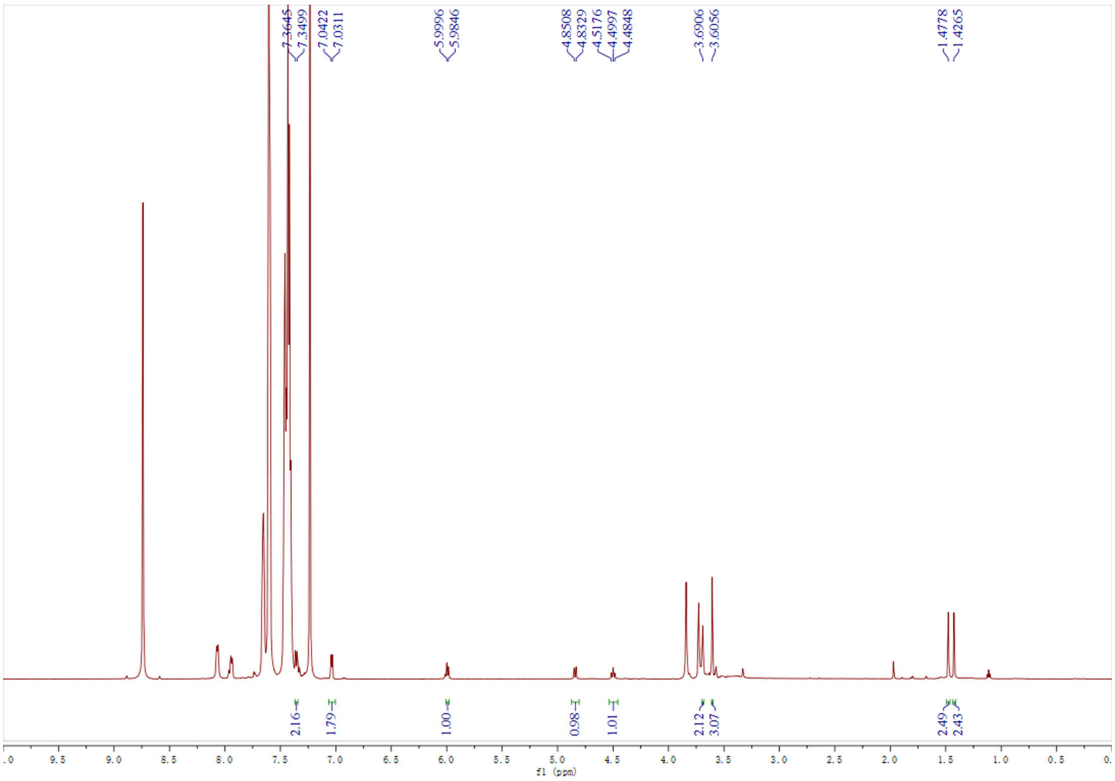

Figure S17. ^{1}H NMR spectrum of (*S*)-MTPA ester of **1b** in Pyridine-d_5 (600 MHz).
